# Synthesis and implication of novel poly(acrylic acid)/nanosorbent embedded hydrogel composite for lead ion removal

**DOI:** 10.1038/s41598-017-15642-9

**Published:** 2017-11-27

**Authors:** Mayuri Bhatia, Satish Babu Rajulapati, Shirish Sonawane, Amandeep Girdhar

**Affiliations:** 1 Department of Biotechnology, National Institute of Technology Warangal, Warangal, 506004 Telangana India; 2 Department of Chemical Engineering, National Institute of Technology Warangal, Warangal, 506004 Telangana India; 3 Department of Biotechnology, Indian Institute of Technology Hyderabad, Sangareddy, Kandi, 502285 Telangana India

## Abstract

Lead stands second among the deadly heavy metal pollutants owing to the incompetent mechanism possessed by the human body for its removal. A polymeric hydrogel in the form of composite was prepared using acrylic acid (monomer) and novel nanofiller that possess super adsorbent properties with restricted gel seepage into flowing ionic liquid. The filler used is an adsorbent which is biocompatible, biodegradable, economical, abundant, non-hazardous and easy to synthesize. The invariably porous nanofiller, the Nanobentonite(clay), was synthesized using ion exchange reaction by creating acidic environment for accelerated dispersion with exfoliation by CTAB to enhance cation exchange capacity. NanobentoFnite was capable of removing >97% lead ion in batch adsorption study and followed pseudo-second order kinetic model. Freundlich isotherm suggested a removal capacity of ~20 mg/g. Thus, the successfully experimented adsorbent was implicated as filler to form polyacrylic acid nanoclay hydrogel polymerized in ultrasonic bath. The amount of filler was varied from 0.25 to 2 wt% to get 94% removal, analyzed using ICP-OES. The prepared adsorbents were characterized before and after adsorption using TEM, FESEM, XRD, FTIR and DSC to understand the structural changes and metal-sorbent interaction. Thus, the novel nanosorbent/composite are promiscuous and competent in terms of availability, reusability and longevity to remove heavy metal ions.

## Introduction

Exposure to lead can be waterborne, airborne or food borne, where lead escapes into water usually due to leakage in pipes^1–3^. High water temperature, acidity and standing time in corroded and non-corroded pipes are major sources including cracks in solder and fittings that leads to lead seepage. It can’t be absorbed by body, so using lead contaminated water for other households is manageable. It mostly enters the human body through food or drinking water. Infants are more vulnerable to bioaccumulation of lead due to active development and also, more consumption in liquid form^4,5^. Body lacks suitable mechanism for clearance of accumulated lead. Thus, it is important to remove lead from natural source, water and concentration as low as 10 µg/L is enough to be hazardous^4,6,7^.

There are many physical, chemical and biological ways being explored to overcome pollutant contamination. The list mostly comprise of reverse osmosis, ion exchange, UV treatment, ozonization, activated carbon purifiers, micron filters, adsorption etc. The present work has been concentrated on the cost-effective and efficient treatment incentive i.e. Adsorption^8–10^. Adsorption can be performed biologically, chemically and in form of composites^11–15^. Nanotechnology has found its emerging implementation for wastewater treatment based on adsorption principle. The high throughput efficiency of nanoparticles accounts to high surface area, more adsorption sites, adsorption specificity, high permeability, high chemical stability, lower toxicity and cost in comparison to bulk particles that result in improved efficiency and longevity^10,16–23^. In the present investigation, bentonite clay nanoparticles (aka nanoclay) were prepared using ion exchange mechanism. Bentonite is an abundantly found clay mineral that has high water adsorption, cation-exchange and ionic substitution abilities. Clay has been used since antiquity for various applications including organic synthesis, food additive, antibacterial activity, mineralogical attributes, adsorbent for ionic dyes, etc^24^. Thus, Nano- form of bentonite clay will have improved cation ion exchange capacity, hydrophilicity, absorption capacity and interactive functional groups on surface with increased surface/volume ratio due to nanosized particles^25,26^.

Another material gaining attention of the researchers is super adsorbent hydrogel. These hydrogels are made from different synthetic and/or natural polymers that have been tested successfully to remove organic and inorganic pollutants^27,28^. Polymeric hydrogels comprise of high internal crosslinking that provides voids and sorption sites for achieving maximum adsorption^29^. Polymers have found vivid applications in the field of science ranging from medical application for controlled drug release to environmental application in the form of biodegradable plastics and adsorbents to food industries as raw material (eg. Starch)^30,31^. Besides, the list of industrial applications of polymers is huge as they form an evident source of raw materials owing to their properties. Hydrophilicity, permeability, reusability and ecological fitness are a few to be considered for the extended applicability in water treatment^32^.

These polymer generally are accompanied with low mechanical strength^30^ and easy runway with water stream thus, fillers are added to encroach their strength for stable activity^33,34^. Nanoparticles are mostly implemented as high aspect ratio fillers to improve mechanistic features without hurting the real application^35^.

The present work has explored the application of nanoclay as adsorbent and filler. The physical and chemical parameters influencing adsorption process of nanoclay were optimized (pH, contact time and adsorbent dose) for high end lead sequestration. On achieving successful lead removal the adsorbent was further implicated as filler to enhance the properties of super adsorbent, hydrogel. The nanoclay hydrogel composite, thus formed, had strengthened mechanical properties due to increased crosslinking of nanoclay and polymer. Hydrogel is water soluble and can escape into water with heavy flow due to weak hydrophilic bonding^36^. Supplementing hydrogels with nanosorbent helps to hold the weak bonds along with supportive adsorption. Thus, synthesized nanocomposites were implicated for removal of lead ions to understand the adsorption potential of gel composite on addition of filler.

## Results

### Characterization

**Figure 1:**
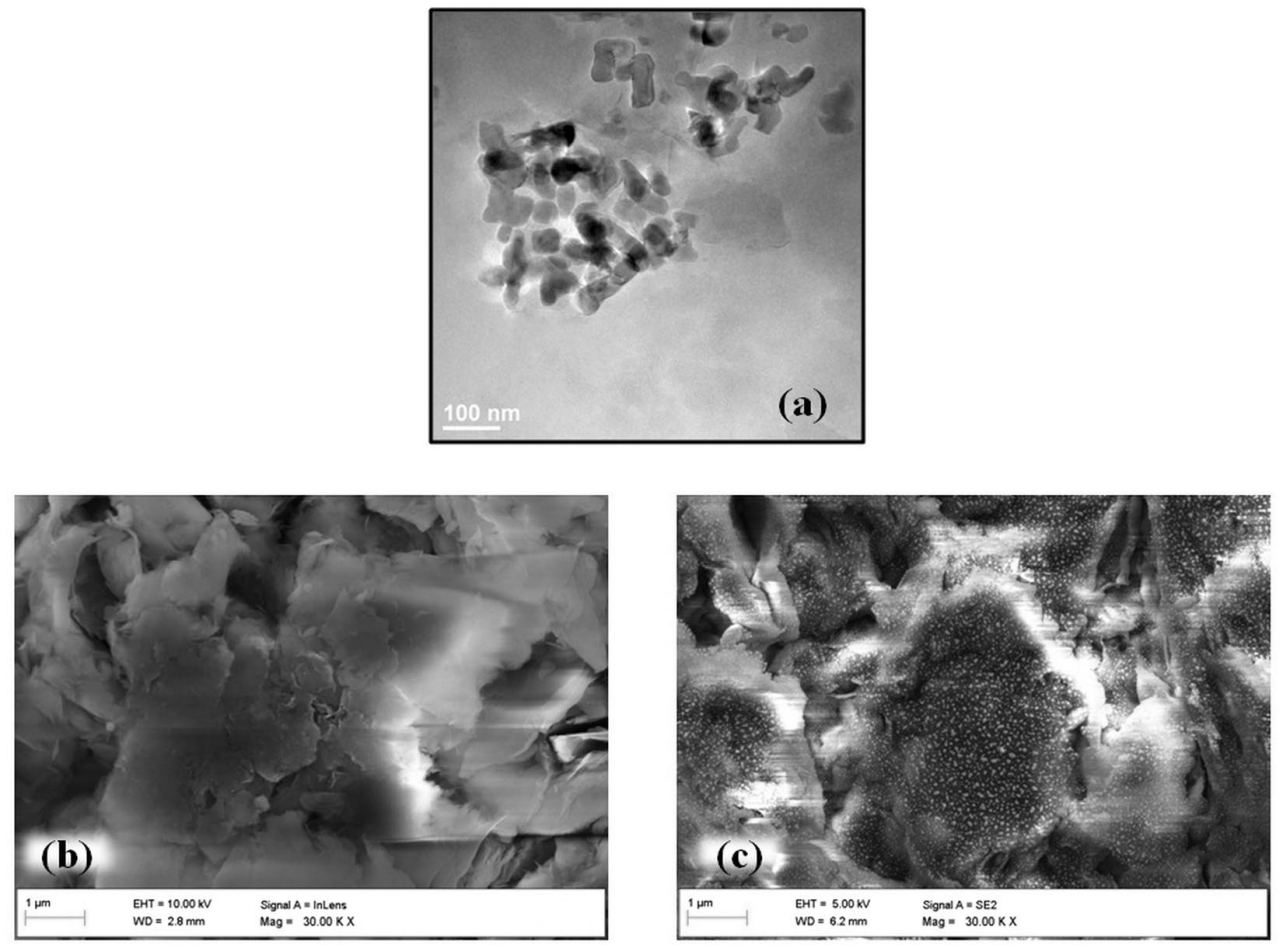
(**a**) TEM image of nanosized clay particles and FESEM image of nanoclay (**b**) before and (**c**) after adsorption of lead ions.

**Figure 2:**
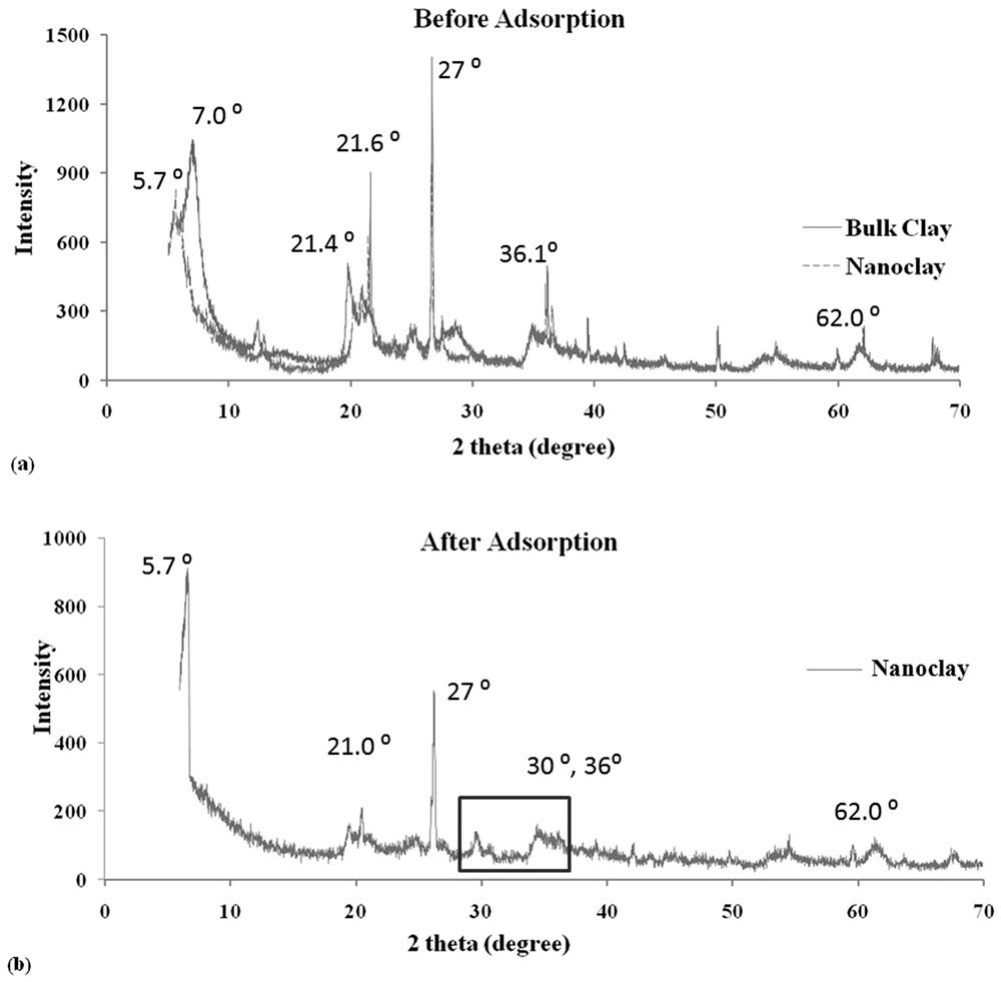
XRD pattern of nanoclay (**a**) before adsorption depicting characteristic peak overlay of bulk and nanoparticles of bentonite clay corresponding to montmorillonite group and (**b**) after adsorption depicting montmorillonite characteristic peaks with peaks of lead ions (highlighted at 30° and 36°).

The nanosized particles of clay were observed using Transmission Electron Microscopy (TEM), as shown in Fig. 1(a). The size of bentonite particles was less than 100 nm. The surface morphology was characterized by FESEM that displayed porous and layered morphology for nanoclay (Fig. 1(b)). Similar morphology has also been reported by^37^. Energy Dispersive X-ray gave elemental composition of nanoclay with 37.3% oxygen, 12.12% aluminum, 29.58% silicon, 0.34% magnesium and 1.78% iron. Figure 2(a) evidently showcases characteristic peaks at 6.8°, 21°, 36° and 62° for montmorillonite group, as bentonite (here nanoclay) and 27°, for quartz, also reported by^37–39^. Further, the sharp peaks are resultant of crystalline nature of nanoclay and a reduced intensity of nanoclay peaks in comparison to bulk clay accounts to the formation of particles of smaller size. These 2 θ values were comparable with the characteristic values of bulk clay XRD pattern as shown in Fig. 2(a) and Table 1. The increase in gallery spacing was observed from 12°A to 15°A, whereas the average crystal size reduced from 12.93 nm to 9.46 nm, as calculated using equations 1 and 2, respectively.

**Table 1:** 2 theta values obtained from XRD pattern with corresponding intensities and gallery spacing (d) for bulk clay and nanoclay.

Bulk Clay			Nanoclay		
2 theta	Intensity	d (°A)	2 theta	Intensity	d (°A)
**7.007032**	**1047**	**12.6**	**5.625349**	**827**	**15.69**
21.61268	904	4.11	21.41215	623	4.14
36.15148	496	2.48	36.03451	309	2.49
62.13751	236	1.49	62.02053	229	1.5

**Figure 3:**
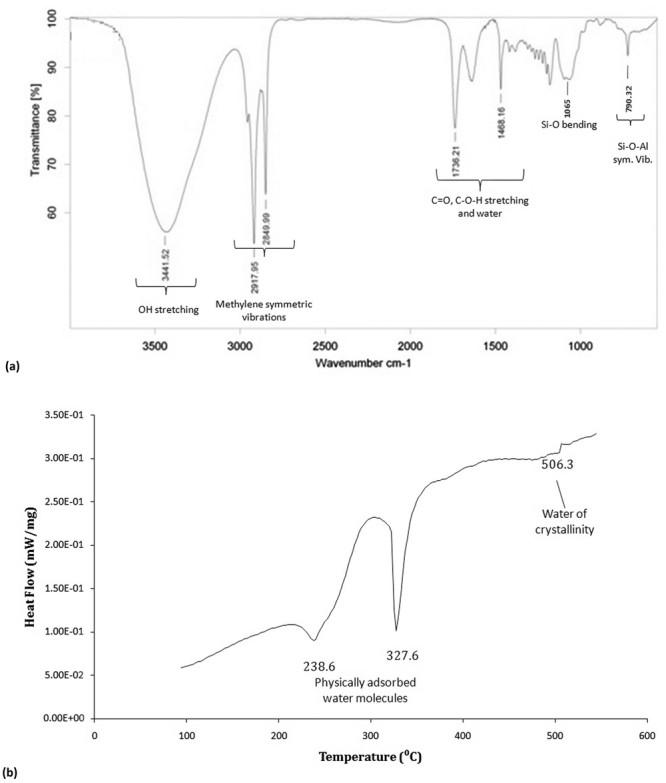
FTIR spectrum of (**a**) nanoclay with marked functional groups present on the surface, involved in adsorption with characteristic stretching for Al-Mg-OH and Si-O and (**b**) DSC thermogram of nanoclay.

The functional groups present on the adsorption sites influence the binding of heavy metals. Thus, to know the surface functionality Fourier Transform Infrared (FT-IR) Spectroscopy was performed for nanoclay, hydrogel and composites. The FT-IR spectrum of nanoclay gave OH- stretching, methylene symmetric vibrations, C=O, C-O-H and Si-O bending, Al-Mg-OH bending and Si-O-Al symmetric vibrations between 3600–3300 cm^−1^, 3000–2600 cm^−1^, 1800–1000 cm^−1^, 1000–800 cm^−1^ and 800–600 cm^−1^, respectively (Fig. 3(a)). The bentonite fingerprint spectrum has also been reported by^37,40,41^.

**Figure 4:**
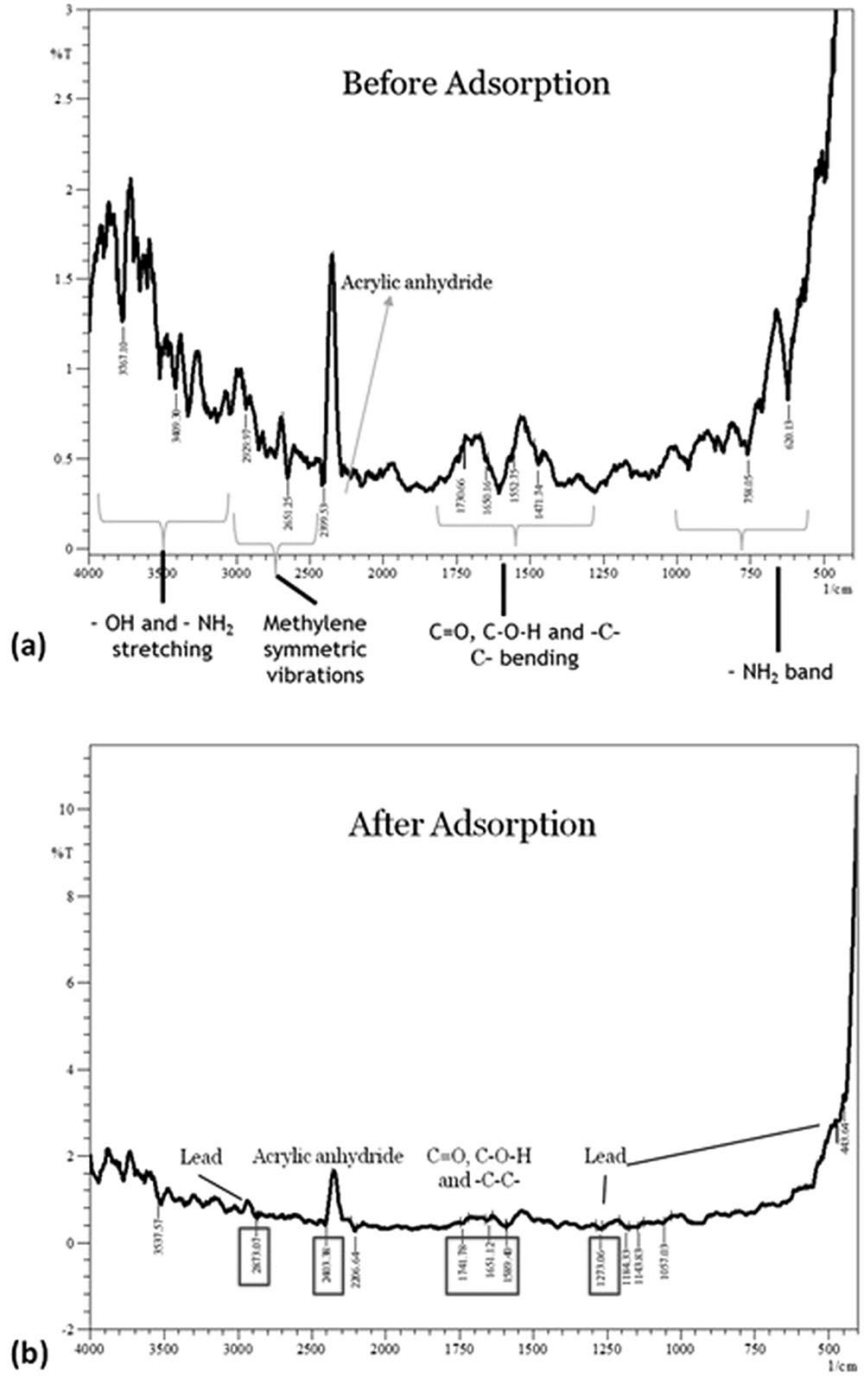
Fingerprint FTIR spectrum of (**a**) hydrogel before adsorption and (**b**) after adsorption lead removal, confirmed by lead characteristic peaks observed at 2873, 1273 and 443 cm^−1^.

Hydrogel FTIR spectrum depicts characteristic peak for polymeric groups and acrylic anhydride, including other regions. Peaks within 3700–3000 cm^−1^ corresponds to –OH and –NH_2_ stretching, 3000–2000 cm^−1^ includes methylene aliphatic and aromatic vibrations including peak for acrylic anhydride, followed by C=O, C-O-H, -C-O-C- and -C-C- bending between 1800–1100 cm^−1^ and finally, amide band for <1000 cm^−1^. The major peak of acrylic anhydride was prominent at 2399.53 cm^−1^; and peaks for polymeric groups (C=O, -C-O-H and -C-C-) at 1730 cm^−1^, 1650 cm^−1^, 1552 cm^−1^ (Fig. 4(a)).

**Figure 5:**
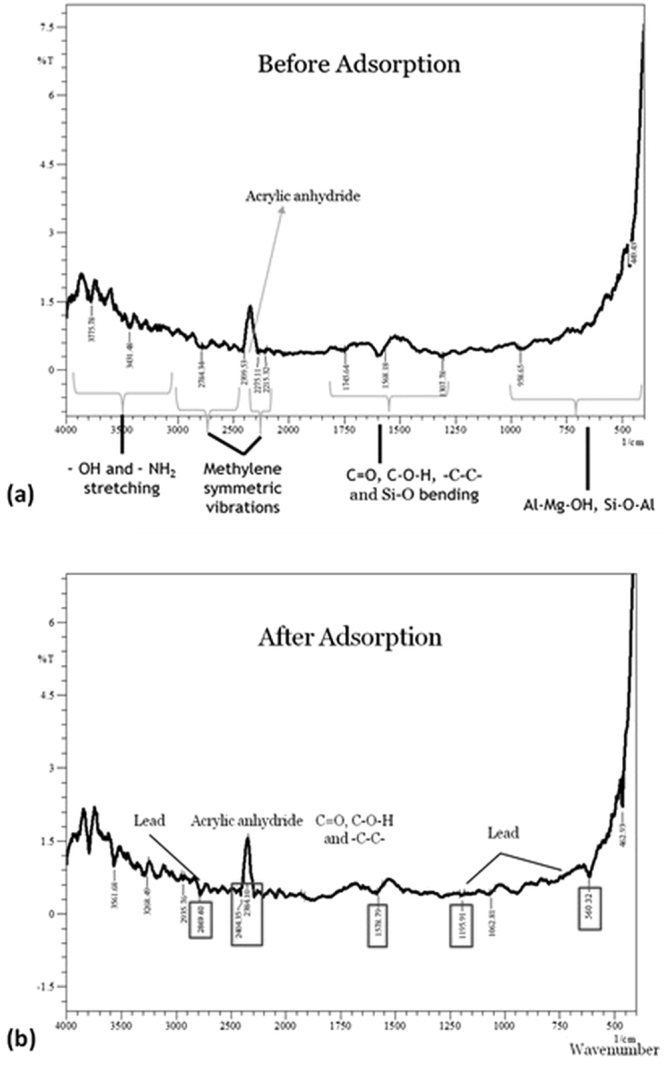
Fingerprint FTIR spectrum of nanocomposite with characteristic peaks for hydrogel (acrylic anhydride) and nanoclay before adsorption (**b**) after adsorption of lead removal confirmed by lead characteristic peaks observed at 2873, 1195, 560 and 462 cm^−1^.

In nanoclay hydrogel, before adsorption FT-IR spectrum is quite evident of the similar peaks as in hydrogel with a few additions in the range of 400–1000 cm^−1^ that are attributed to Al-Mg-OH, Si-O-Al groups. These groups are characteristic for montmorillonite/bentonite clay (Fig. 5(a)). Band for acrylic anhydride was observed at 2399.53 cm^−1^ that confirms the polymerization.

Thermal analysis of nanoclay was performed using Differential Scanning Colorimetry (DSC). DSC shows two peaks at 238.6 °C and 327.6 °C for removal of adsorbed water molecules, whereas peak at 506.3 °C shows the escape of water of crystallinity (Fig. 3(b)).

### Optimization study for Nanoclay

Optimization of physical and chemical parameters was performed by batch adsorption studies. The batch adsorption was carried out in shake flask by monitoring one parameter at a time to achieve maximum removal efficiency.

#### pH

Lead removal is completely dependent on pH of the solution. The experiment was set with negative controls including nanoclay in distilled water and lead solution without adsorbent at variable pH. The pH was varied from 2–10, where pH 6 was found to be optimum and above pH 6 i.e. at pH 7 and 8 a slight turbidity in the solution can be seen whereas, further increase in pH to 9.0 ± 0.2 and 10.0 ± 0.2, clear precipitation of lead hydroxide salt occurs. The variation in the turbidity can be clearly seen in Supplementary Figure S1. Therefore, the working pH was selected as 6.0 ± 0.2, succeeding which the removal wasn’t solely a result of absorption. At alkaline pH, lead started forming hydroxide which is insoluble in water and thus, precipitates at the bottom. Precipitation of lead ions has also been reported by Salam *et al*.^42^. Moreover, it has been stated that alkaline pH accelerates the lead hydroxide formation^42,43^. Thus, above pH 6 the sequestration is not solely dependent on binding ability of adsorbents, but because of precipitation. The adsorbent dose was kept constant at 3 g/L with 24 hours incubation. The maximum removal at pH 2 was <5% that increased to 77% at pH 6.00.

**Figure 6:**
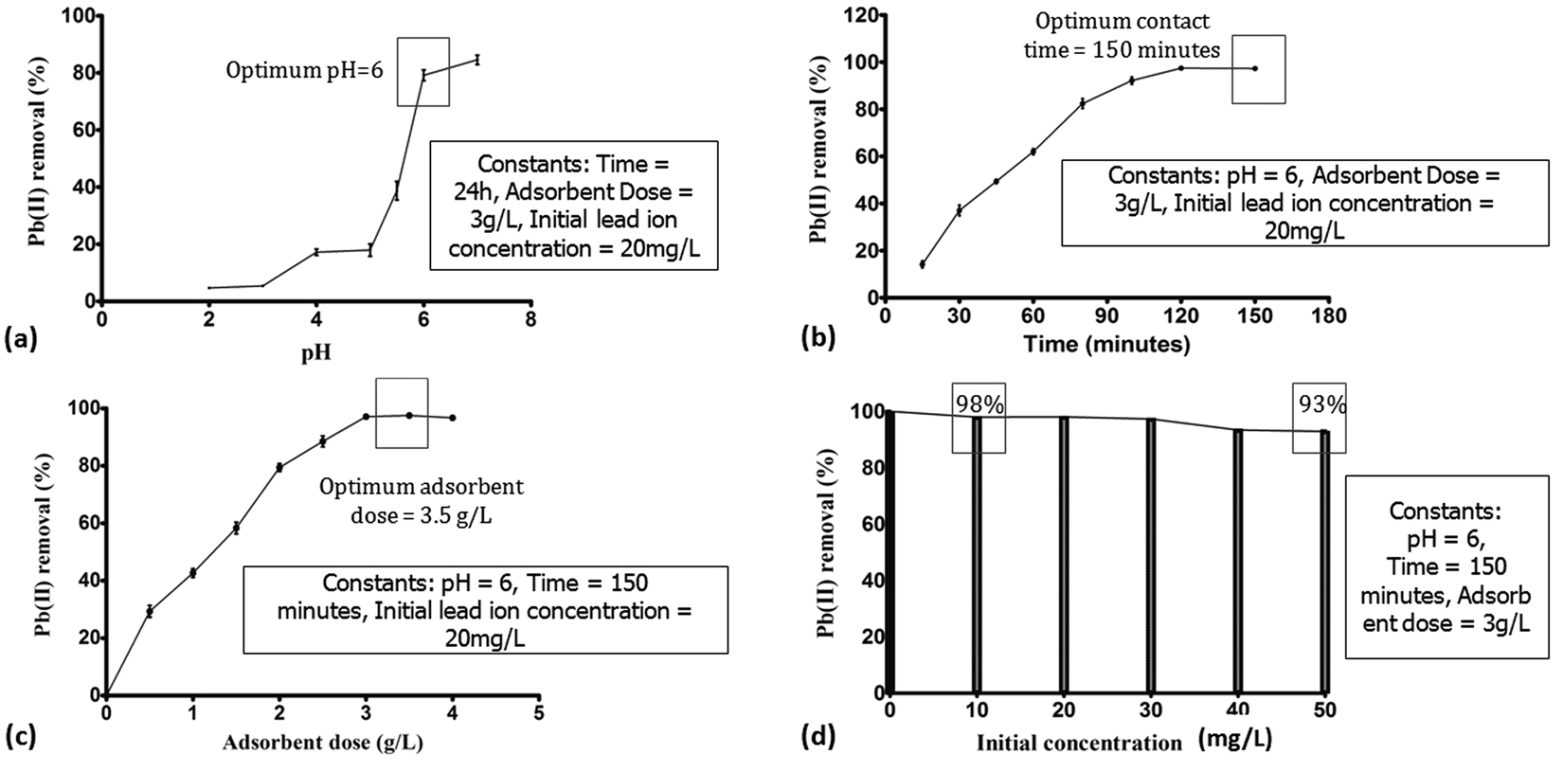
Optimization data for nanoclay (**a**) pH, (**b**) contact time, (**c**) adsorbent dose and (**d**) initial lead ion concentration; Error bars represent standard deviation from three independent samples (n = 3, P < 0.01, One way ANOVA).

Lead is present in cationic form in the solution, thus, competes with H^+^ for active adsorption sites. The acidity of solution decides the sequestration. If the solution is more acidic, it indicates the presence of H^+^ in higher concentration that limits lead ion binding. On the contrary, basic pH indicates presence of OH^−^ that allows binding of lead cations^44^. The other mechanism responsible for the adsorption is functional group sequestration by surface complex formation. Figure 6(a) describes the pH dependent binding for nanoclay. Even adsorption efficiency can be influenced by altering the surface groups^44^. At acidic pH, columbic repulsions are countered by lead ions, whereas basic pH leads to columbic attractions.

#### Contact time

The nanoadsorbent was left in the solution till the percent removal becomes constant. Samples were collected at regular intervals of 30 minutes. Nanoclay was found to give effective removal of >95% in 150 minutes. The adsorption was fastest in first 60 minutes due availability of active sites in huge number thus; adsorption is at its peak. Gradual filling of active sites lead to slower adsorption. It also depends on intraparticle diffusion. The increase in adsorption with changing pace for nanoclay can be seen in Fig. 6(b). In earlier studies, the equilibrium was reached in 300 minutes and 120 minutes respectively, for adsorption by chitosan clay and carbon nanocomposites^42,45^.

#### Adsorbent dose

Adsorbent dose was varied from 0–4 g/L in both the cases. Figure 6(c) depicts the variation in lead removal with increase in adsorbent dose. 0.5 g/L of nanoclay gave ~30% sequestration that increased to 97.5% with 3.5 g/L adsorbent dose. More the adsorbent more is the adsorption due to increasing number of active sites. But gradually these adsorption sites begin to overlap and results in reduced active sites per unit mass. Also, due to agglomeration, nanosized particles start behaving like bulk particles. On increasing nanoclay dose from 3.5 to 4 g/L, a reduction in adsorption was observed which can be clubbed with the fact of site saturation. Adsorption remains unaffected on increasing the adsorbent dose above 3.5 g/L which may be due to agglomeration of nanoparticles, ultimately masking many available or unsaturated adsorption sites. The phenomenon of nanoparticle agglomeration has also been reported by a study performed using iron oxide nanoparticle for lead removal that explains the significance of optimized dosage^46,47^. For nanoclay, the optimized adsorbent dose at optimized pH and contact time was 3.5 g/L and maximum removal achieved is 97.5% when lead concentration was 20 mg/L.

#### Initial metal ion concentration

Initial lead concentration for previous experiment sets was kept constant at 20 mg/L. Effect of initial lead ion concentration was studied next on the optimized parameters, especially adsorbent dose. Figure 6(d) displays the reduction in efficiency of adsorbent on increasing metal ion concentration. When lead concentration was as low as 10 mg/L, removal efficiency of nanoclay was 98% that decreased to 93% on increasing metal ion concentration to 50 mg/L. Thus, initial metal ion concentration is a primary factor to decide the amount of adsorbent to be added to attain maximum possible removal. The adsorbent dose has to be optimized for each adsorbate depending on the amount of metal ion present. The influence of initial lead ion concentration over the adsorption kinetics has also been observed by Rajput *et al*.^47^.

### Adsorption Kinetics

**Figure 7:**
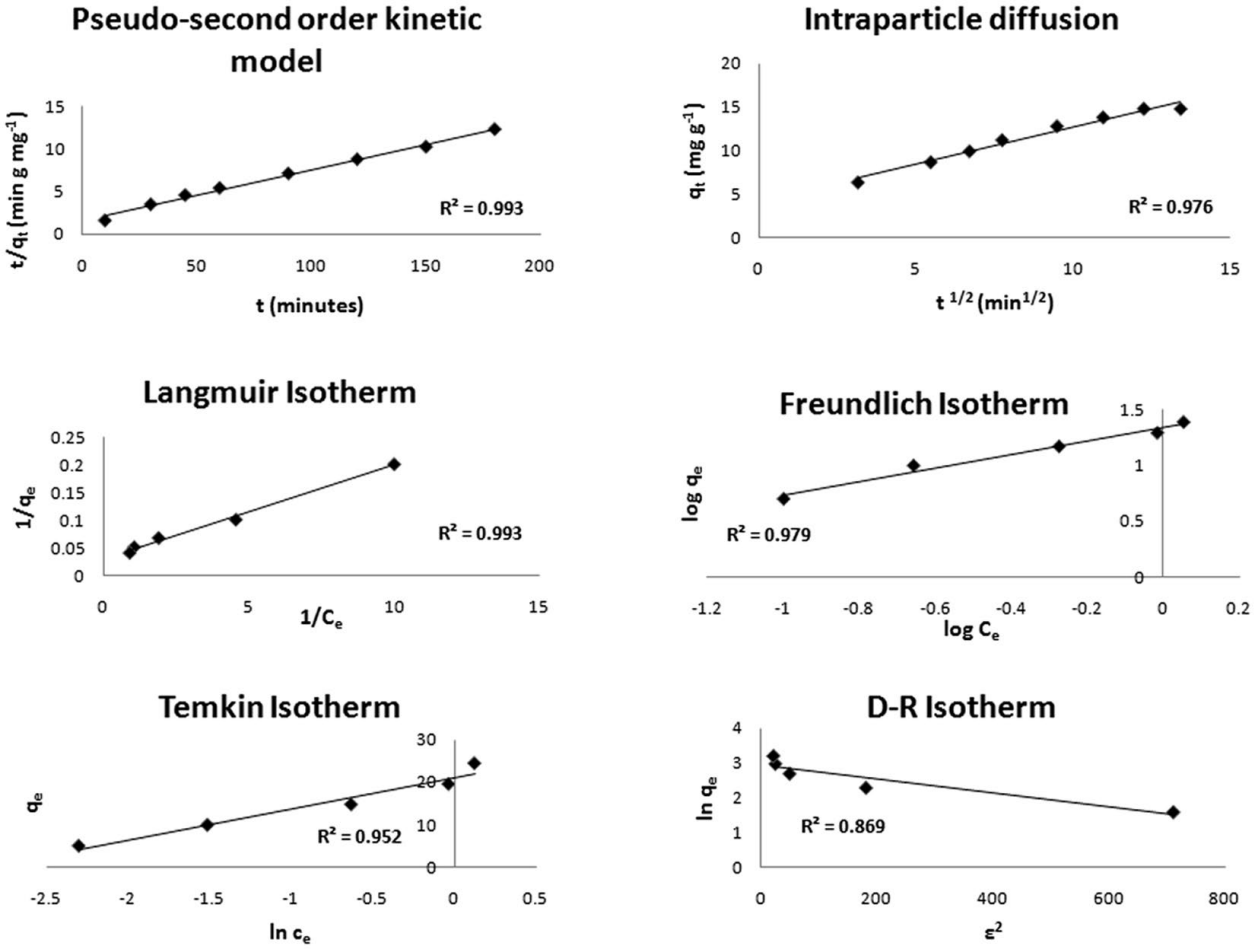
Graphs depicting adsorption kinetics performed using Pseudo-second order and intra particle diffusion kinetic models and the adsorption isotherms, Langmuir, Freundlich, Temkin and D – R isotherms studied.

The prepared nanoclay was found to be competent enough for removing  lead ions and thus, studied further for adsorption kinetics. The graph was plotted between log (q_e_ − q_t_) and t to determine the rate kinetics of the adsorption study conducted for nanoclay. The graph has been depicted in Supplementary Figure S2 (supplementary data), where R^2^ value is 0.994 with experimental q_e_ (15 mg/g) is near to theoretical q_e_ calculated (10.89 mg/g) from the plotted equation with an approximate error percentage (APE) of 5.6. Therefore, the adsorption study defies pseudo-first order kinetic model (Table 2). The pseudo-second order kinetics was next checked and the graph plotted has been shown in Fig. 7. The regression was 0.993 with q_m_ 16.95 mg/g and k_2_ 0.006 g/mg min. APE in this case was 0.5 that was lesser than former kinetic model and thus, pseudo-second order was taken as best fit. For intra-particle diffusion study, the constant k_id_ was 0.849 mg/g min^1/2^ and R^2^ was 0.98. The adsorption process thus, follows chemical adsorption assisted with ion exchange and surface complexation as suggested by pseudo-second order kinetics and also, was found to be in agreement with intra-particle diffusion kinetics denoting the participation of each nanoparticle for diffusive interaction with lead ions (Fig. 7). The kinetics shown by the adsorption study is in line with the study performed on anatase and also, by bentonite adsorbent for lead and arsenic removal, respectively^48–50^.

**Table 2:** Kinetic model with respective parameters.

Kinetic Model	Pseudo-first order				Pseudo-second order				Intra-particle Diffusion		
Parameters	k_1_ (min^−1^)	q_e_ (mg g^−1^)	R^2^	APE	k_2_ (gmg^−1^ min^−1^)	q_e_ (mg g^−1^)	R^2^	APE	k_id_ (mg g^−1^ min^−1/2^)	R^2^	APE
	0.018	10.89	0.99	5.6	0.006	16.95	0.99	0.5	0.849	0.98	13.6

### Adsorption Isotherms

The effective adsorption depicted by nanoclay was subjected to isotherm analysis, where the optimization data was analyzed using four different isotherms namely, Langmuir, Freundlich, Temkin and Dubinin-Radushkevich (D-R) models. The graphical representation has been shown in Fig. 7 and the corresponding values of different parameters are given in Table 3. The maximum adsorption given by Langmuir isotherm is 33.33 mg/g which is quite exceeding the experimental value (15 mg/g) though regression coefficient was quiet high and its value was 0.99. On calculating the separation factor, R_L_ it was further confirmed that the isotherm is an unfavourable fit, as R_L_ value exceeded 1. R_L_ was calculated using equation 8. Freundlich model, on the other hand gave comparable adsorption value as 19.95 mg/g (K_F_) with regression of 0.98. The model is favorable for the adsorption as the slope; 1/n is 0.607, lying between 0 to 1. The next isotherm that denotes adsorption intensity and capacity is Temkin model, where maximum calculated adsorption was 17.85 L/g (K_T_). The heat of adsorption was 7.317 KJ/mol which was comparable to previous studies performed using novel silica based hybrid adsorbents for lead removal, dye removal using nano-geothite and also, for biosorption using peanut husk. The heat of adsorption can vary from system to system and gives a hint about the nature of adsorption process. A low value for b_T_ (0.34 kJ/mol, in present study) defines weak physical and stronger chemical bonding between adsorbent and adsorbate^48,51–53^.

**Table 3:** Adsorption Isotherms with respective parameters.

Langmuir			Freundlich			Temkin			D-R			
q_m_ (mg g^−1^)	K_L_ (L mg^−1^)	R^2^	K_F_	1/n	R^2^	K_T_ (L g^−1^)	b_T_ (kJ mol^−1^)	R^2^	q_m_ (mg g^−1^)	β	E_a_	R^2^
33.33	1.76	0.99	19.95	0.607	0.98	17.85	0.34	0.96	18.95	0.002	15.81	0.9

Subsequently, Dubinin-Radushkevich (D-R) isotherm was applied to determine the mean free energy of the process. The maximum adsorption calculated by the model is 18.95 mg/g with mean energy constant value as 0.002 mol^2^ kJ^−2^. Further, the constant was placed in equation (13) to calculate mean free adsorption energy and was observed to be 15.81 kJ mol^−1^. The mean energy value lying between 8–16 kJ mol^−1^ is said to follow chemical adsorption involving ion exchange and the value less than 8 kJ mol^−1^ is denoting physical process^48,51,52^. The former process stands true for current study i.e. chemisorption. Freundlich, Temkin and Dubinin-Radushkevich isotherm models basically stands true for a non-uniform surface, implicated in the adsorption phenomenon observed for nanoclay.

### Thermodynamics

The spontaneity of the adsorption process followed by nanoclay was analyzed by Gibbs free energy using equations 14 and 15. The K_C_ was found to be 27.81 L/g and on its implementation the ∆G° was calculated as −8.24 kJ/mol. The negative value stands for the feasibility and spontaneity of the adsorption process. The higher negative ∆G° values denote higher process spontaneity^48,50^.

### Application of nanoclay as filler: Hydrogel composites

The application of nanoclay as filler was studied to overcome the limitations of using PAA hydrogel as adsorbent. PAA hydrogel is a well known super adsorbent with efficient role in dye removal with a fewer metal removal based implications. The current work has tried to explore hydrogel as adsorbent for metal ion removal. On performing batch sorption study on prepared PAA hydrogel, >97% lead removal was attained in 105 minutes at pH = 6.00 and flow rate 1.3 mL/sec. The hydrogel efficiently removes lead ion over growing contact time and then becomes stable after saturation of available adsorption sites. Hydrogel has been reported to give more than 96% dye removal by^54^. Hydrogel has greater water absorption ability and hydrophilicity to interact well with metal ions. Pure hydrogel is quite water loving and can get easily solubilized in water and while the water is flowing with certain pressure, chances of its leakage into treated water is possible.

Thus, to make hydrogel usable as adsorbent for removal of metal ions from water, some binding component is required to be added. The binding component will keep the chemical groups of hydrogel intact and less available for solublization in water. The composites were thus prepared by packing nanoclay into hydrogel. The addition of filler or crossliker improves mechanical strength that has also been proved by Abdel-Halim *et al*.^55^, where starch graft was used as crosslinker. The main aim of the study is to provide economic nanobased filler for increased potential without compromising on the adsorptivity of hydrogel.

**Figure 8:**
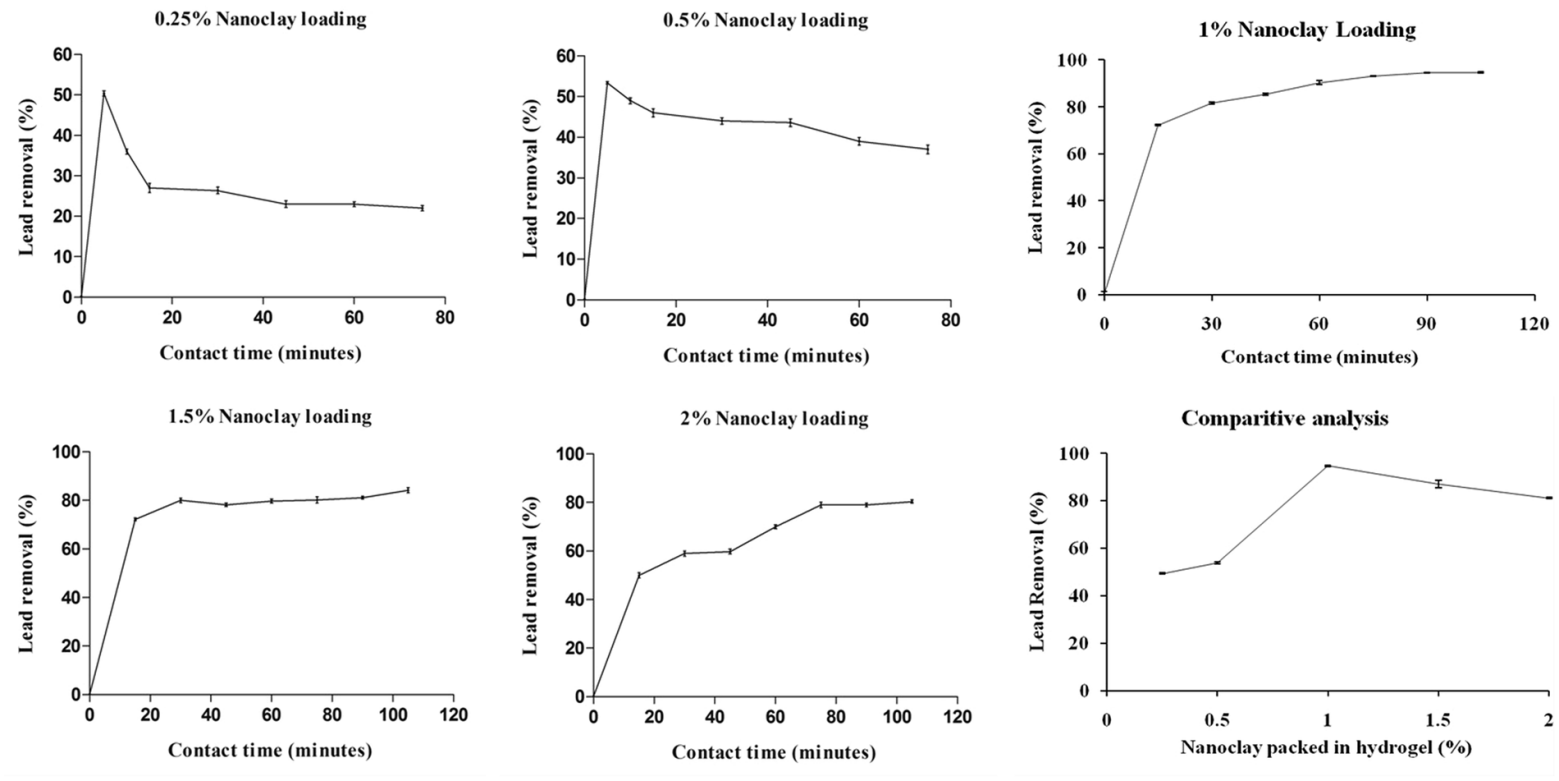
Lead removal by (**a**) 0.25%, (**b**) 0.5%, (**c**) 1%, (**d**) 1.5% and (**e**) 2% nanoclay packed by weight in hydrogel. (**f**) shows comparative analysis between removal efficiencies of different nanoclay loading in hydrogel with maximum removal of 94.6% for 1% nanoclay loading; Error bars represent standard deviation from three independent samples taken at each time frame (n = 3, P < 0.01, One way ANOVA).

The earlier tested potent nanoadsorbent, Nanoclay, was used to synthesize hydrogel composites. The composites made with hydrogel were tested of adsorption potential by varying the sorbent loading from 0.25 to 2 wt%. 10 g of each nanocomposite was used for removal of lead keeping flow rate and pH constant. Nanoclay was appropriately exfoliated in the nanocomposite due to distinctively nanosize particles. Absorption cycle of nanocomposite can be seen in Fig. 8. Nanocomposite with 1% loading gave >94% removal efficiency, which was maximum and decreased to 80%, on increasing the nanoclay loading in hydrogel 2% loading. Increased loading of nanosorbent in hydrogel ensured high adsorbent dosage that resulted in the reduced porosity due to agglomeration of nanosized particles and less availability of active sites on the surface of the composite.

Another observable fact was less adsorption and fast desorption for lower nanoclay loading i.e. for 0.25% and 0.5%. The lesser holding capacity is due to temporary interaction between adsorbent and adsorbate. The complex formation during interaction is not stable thus, reaches equilibrium rapidly leading to a quick desorption. The outlay of the nanocomposite formation is evidently depicted in Supplementary Figure S3, in the supplementary file, with clear notion of crosslinks formed by nanoclay with distinctive layers. These distinctive layers were formed due to proper exfoliation of clay while synthesis. The hydrogel and hydrogel nanoclay composite were experimentally found to give an equivalent adsorption capacity of 9.77 mg/g and 9.73 mg/g, respectively. The respective kinetics of hydrogel and hydrogel nanoclay composite has been graphically presented in Supplementary Figure S3. Thus, the nanoclay was found to be an efficient filler to increase the binding strength without compromising on the adsorption potential of hydrogel.

### Characterization after adsorption

#### Nanoclay

FESEM results are quite evident of presence of lead. Figure 1(c) depicts white dots attached to nanoclay surface, modifying its usual morphology. The used nanoclay was next characterized by XRD. Figure 2(b) depicts the after adsorption XRD pattern that gave new peaks which mostly correspond to lead. X’Pert High Score software was used to analyze XRD peaks corresponding to theta values and intensities. The analyzed data displayed presence of lead at 30° and 36°. Further, lead nitrate XRD spectrum was compared to nanoclay XRD spectra before and after adsorption. In general, 2 theta values that represent nanoclay are 5.6°, 35°, and 62°, whereas, peaks at 31.37°, 36.36°, 52.31°, 62.27° and 65.34° corresponds to lead nitrate. After adsorption data depicts a high angle shift in 2 theta values from 5.6° to 6.7°. The change in the gallery spacing is due to adsorption, which is observable by the high angle shift in the reported pattern. Also, slight peaks have been observed for lead at 30° and 36°. Lead peaks were not very prominent because of fairly less concentration in the sample. Lead XRD pattern has been reported by^56^ and^57^. Thus, it confirms the presence of lead on surface of nanoclay.

#### Hydrogel and its composites: FTIR

Hydrogel and the nanoclay hydrogel composite were analyzed by FT-IR after completion of adsorption process. The used hydrogel and composite were dried and characterized as shown in Fig. 4(b). The FT-IR spectrum for used hydrogel depicts shift in all the major characteristic groups that fairly include C=O, -C-O-H and -C-C- and acrylic anhydride with inclusion of lead fingerprints. Initially in Fig. 4(a) i.e. naïve hydrogel acrylic anhydride was depicted by at 2399.53 cm^−1^ which shifted to 2403 cm^−1^ and peaks corresponding to polymeric groups (C=O, -C-O-H and -C-C-)shifted to 1741 cm^−1^, 1651 cm^−1^ and 1589 cm^−1^. Further, lead peak inclusions were observed at 2873 cm^−1^ and 1273 cm^−1^. Thus, it is clear from Fig. 4(b) that lead was adsorbed on hydrogel.

In case of used nanoclay hydrogel, FT-IR spectrum was quite evident of combinational peaks for hydrogel and nanoclay. Figure 5(b) showcases the peak shifting for acrylic anhydride from 2399.53 to 2404.35 cm^−1^ and for polymeric chain peak from 1568.18 to 1578.79 cm^−1^. Lead peaks were witnessed at 2869.60 cm^−1^, 1195.91 cm^−1^ and 560.32 cm^−1^, which further verifies the adsorption kinetics (Fig. 5(b)).

### Multisorption analysis

The nanoclay hydrogel composite was found to be quite selective for lead adsorption. The adsorption potential of the composite was tested against six metal ions in presence of optimized set of conditions that resulted in maximum adsorption of lead upto 90% in 100 minutes. The efficiency was decreased by only 4% in comparison to single component analysis of lead ion. The multisorption in presence of Cd, Cu, Co, Cr, Ni and Pb depicts inhibition in uptake rate for other divalent ions by lead due to higher ionic radius and electronegativity^58^. Nickel gets precipitated at pH above 5.0 so doesn’t adheres to the adsorbent surface^59^, whereas, copper gives better adsorption at ~pH 6.5, thus, the metal uptake is mostly pH dependent. The optimized parameters were specific for lead; hence nanoclay hydrogel composite gave maximum adsorption followed by copper (accounting to ~25% removal) due to the near optimum pH of the solution. Therefore, the presence of more carboxyl groups on the polymeric gel which gets deprotonated due to effect of pH^34^, and optimized conditions specific for lead removal, the adsorption specificity was attained. The amount of metal removal has been shown in Table 4.

**Table 4:** Metal removal achieved during multi-sorption analysis and the standard deviation is taken from three independent samples for each metal ion (n = 3, P < 0.01; ANOVA: Single Factor).

Metals (20 mg/L)	Adsorption (%)
Cd	4.83 ± 0.4
Co	6.45 ± 0.33
Cr	1.92 ± 0.5
Ni	4.15 ± 0.77
Cu	25.97 ± 0.81
Pb	90.25 ± 0.06

### Desorption and reusability of adsorbents

The reusability of each nanoclay and nanoclay hydrogel composite was tested by a series of desorption and resorption experiments. The adsorbents were saturated with 30 mg/L PbNO_3_ solution at pH = 6.0 ± 0.2 for 24 hours contact time. The adsorbents were washed with distilled water to remove excess unabsorbed lead ions then adsorbent was dried and the sorbent active sites on the surface were regenerated using 0.1 M HCl and 0.1 M NaOH, respectively. Table 5 shows the values of lead desorption obtained in 12 hours. Nanoclay was regenerated up to the capacity of 92% in the first cycle followed by 87.6% site regeneration in second cycle and 76.8% in 3rd cycle using 0.1 M HCl and calculated by equation 16. 0.1 M NaOH on the other hand, was found ineffective for the process and was capable of 48.67% regeneration of nanoclay in first cycle. Thus, was not considered for second regeneration cycle.

**Table 5:** Desorption studies conducted for nanocly and nanoclay hydrogel compoite depicting the corresponding percent desorption in three consecutive cycles. The standard deviation is taken from three independent samples (n = 3, P < 0.01; ANOVA: Single Factor).

Regeneration cycle	Nanoclay			Nanoclay Hydrogel composite		
	Desorbed Lead amount (%) by 0.1 M HCl	Lead adsorption (%)	Desorption %	Desorbed Lead amount (%) by 0.1 M HCl	Lead adsorption (%)	Desorption %
1	89.3 ± 0.32	88.2	92.06 ± 1.64	94.05 ± 0.49	90.2	97.97 ± 1.7
2	84.98 ± 0.7	80.32	87.6 ± 3.62	85.82 ± 0.32	81.5	89.4 ± 1.64
3	74.5 ± 0.2	69.45	76.8 ± 0.09	66.87 ± 0.46	63.65	69.65 ± 2.38

Nanoclay hydrogel was found to be quite effective as it had the potential of regeneration upto 98% in first cycle that lowered down to 89.4% and 69.65% in second and third cycles, respectively. Prolonged exposure of hydrogel in water results in dissolution especially in acidic and alkali conditions that causes sudden fall in active site regeneration. On treatment of Nanoclay hydrogel with 0.1 M NaOH, 13.37% regeneration was achieved in 12 hours and thus, was not considered for further analysis. Alkali solution had an adverse effect on hydrogel leading to an increased solubility rate.

On reusing the adsorbent nanoclay yielded 88% removal after first regeneration cycle and the lead removal reduced to 69.5% in third cycle. Relatively, nanoclay hydrogel gave a removal potential of 90% after first cycle and desorption efficiency decreased to 63% by the end of third regeneration cycle.

## Discussion

Need for water purification technology is ever increasing due to the escalated amount of contaminants in environment. Many countries have banned use of lead containing paints and pigments; though batteries, fertilizers, solder fittings etc. remain the sources of contamination. There are many natural and synthetic adsorbents applied for the removal of hazardous heavy metals. The study here focuses on natural, nano- and composite adsorbents as an environmental friendly alternative. Also, it has discussed the benefits of using nanometer sized adsorbents. Each particle of nanosorbent has ability to perform like bulk particles. Moreover, the increased surface to volume ratio gives better availability of adsorption sites; hence, increased adsorption. Composites are new age adsorbents being implemented for enhanced efficiency and reusability.

The prepared nanoparticles from bulk bentonite clay were characterized by TEM, FESEM, XRD, FTIR and DSC. The assessment supported the nanoparticle formation with a porous morphology that was a resultant of exfoliation of layers in nanoclay. The characteristic montmorillonite peaks remained unaltered with a decreased intensity showcasing smaller particle availability in the sample in comparison to bulk clay particles. Nanoparticle formation was a type of modification caused by treatment with various chemicals including CTAB, SDS, etc. that might enhance or suppress the FTIR fingerprint spectrum without altering it. Thus, the nanoclay FTIR spectrum was compared with bulk clay spectrum which was in agreement. The characterization data was in line with the earlier reported literature^37–39^. Further, hydrogel and nanoclay hydrogel composite were prepared by polymerization reaction carried out in ultrasonic bath and was characterized by FTIR. The acrylic anhydride characteristic peaks confirmed the completion of polymerization in both the cases. The addition of nanoclay peaks in hydrogel FTIR spectrum was evident of composite formation. The peak addition with appearance of characteristic fingerprint region has also been reported by Heidari *et al*.^58^ for chitosan/montmorillonite composite. The prepared naoclay was also found to be thermally stable and degrades completely at 506.3 °C, as tested by DSC.

The nanoclay was experimented as a potent adsorbent and applied as “nanofiller” to remove lead from water and provide binding strength^34^. The parameters influencing the removal efficiency were optimized to gain maximum lead ion sequestration. For the purpose, lead nitrate solution prepared in distilled water was used and the lead concentration was quantified with the help of ICP. The solution pH was found to be most affecting parameter which basically increases adsorption on increasing pH to 6.00 and starts forming hydroxide on reaching pH in alkaline range causing removal by precipitation. The optimized pH 6.00 was considered for contact time optimization where, samples were collected at regular intervals for 24 hours. Rapid adsorption was observed for first 60 minutes with a gradual decrease in rate of adsorption until equilibrium was attained. The contact time study followed pseudo-second order and intra-particle diffusion kinetic models indicating the role of ion exchange due to particulate diffusion and surface complexation reactions to achieve chemical adsorption. The adsorbent dose and initial lead concentration in the pH and contact time study were kept constant, which were next optimized, where, initial lead ion concentration was found to be the major factor in deciding adsorbent dose. Less adsorbent dose can cause less removal, whereas, more adsorbent can also cause less removal due to particle agglomeration that mask the unsaturated sorption sites. The effect of initial lead ion concentration study was further analyzed using adsorption isotherms. Freundlich isotherm followed by the experimental figures denotes a non-uniform adsorption. Temkin and D-R isotherms, on the other hand, supported the experimental kinetics by suggesting strong chemical interaction i.e. chemisorption on to the non-uniform surface. Lastly, the reaction was found to be spontaneous and feasible due to high negative Gibbs free energy.

The high potential of nanoclay was applied as filler to support adsorption by super adsorbent hydrogel. Hydrogel itself has great adsorption ability, however, poor mechanical strength restricts its application. Hence, nanoclay was added as filler that created better crosslinking to not let the gel escape into flowing water. The addition of nanoclay was truly advantageous but has reduced porosity. The amount of nanoclay to be added was optimized where 1% nanoclay filler was found to give maximum removal.

A study has successfully demonstrated synthesizing poly acrylic acid–bentonite nanocomposite in form of particles, whereas the current study has used PAA superadsorbent hydrogel (Figure S2). Table 6 provides an overview of similar studies performed for lead removal. Here, the hydrogel has been impregnated with nanobentonite clay as filler to hold the dissolution of hydrogel in the flowing ionic liquid. Thus, the composite preparation methodology is completely different and is novel and unique. The synthesis of modified bentonite was quite similar, though the outcome of the current study was modified bentonite nanoparticles that were confirmed by TEM analysis. Further, for adsorption study with nanoclay hydrogel composite, packed bed system was used, whereas, the adsorption with modified bentonite i.e. nanoclay, was directly carried out in shake flask. Lastly, the prepared adsorbents, nanoclay, hydrogel and nanoclay hydrogel composite, were characterized by XRD and FTIR, where the characteristic peaks of lead were seen in the spectrum. These peaks were absent before adsorption that proves the adsorption potential of the adsorbents. The confirmation and quantification of lead removal was carried out by analyzing each sample by ICP-OES.

**Table 6:** Comparison of current study with the literature on the basis of preparation methodology, physical and chemical parameters and adsorption capacity.

Nanoparticles	Preparation	pH	Contact Time (min)	Adsorbent Dose (g/L)	Lead concentration (mg/L)	Isotherm and kinetic model	References
Nanoclay	Ion exchange reaction	6	150	3.5	20	98%, Freundlich, Pseudo-second order, 19.95 mg/g	This study
Hydrogel	Polymerization, ultrasonication	6	100	10	100	97%, Pseudo-second order, 9.7 mg/g	This study
Nanoclay Hydrogel Composite	Polymerization, ultrasonication, Stirring	6	100	10	100	94%, Pseudo-second order, 10.1 mg/g	This study
Chitosan/Clay	Activated nanoclay was added to solution containing chitosan.	6	300	6	50	Upto 80%, Freundlich isotherm (0.7 mg/g) and pseudo-second-order kinetics	45
Bentonite particles	CTAB exfoliation	6	1440	0.35	800	Freundlich, 15.39 mg/g	64
poly(acrylic acid)/bentonite nanocomposite	Intercalation	6	30	7.5	400	Freundlich, 33.77 mg/g	64
NanoBentonitecrosslinked Chitosan	Activated clay added to chitosan:acetic acid solution and crosslinked using glutaraldehyde	4.5	80	0.5	1000	Langmuir Isotherm (7.93 mg/g) and thermodynamic parameters were studied	66
ZnO montmorillonite composite	Green simple heat	4	75	60	100	Freundlich, 26.41 mg/g	67
Chitosan/methacrylic acid (MAA)	Polymerization of MAA in chitosan solution, followed by freeze drying	6	120	5	20 × 10^3^	Langmuir isotherm (13.72 mg/g) and pseudo-second- order kinetic model was followed	58
Anatase	Sol-gel method using tetraisopropoxide and 2-propanol	6	750	0.015	10	Langmuir model (31.25 mg/g)and followed pseudo-second order kinetics	49
TiO_2_	Commercially available	5	60–90	2	0.1	Isotherm not applicable but followed first-order kinetic model (21.7 mg/g)	44
Superparamagnetic maghemite (γ-Fe2O3) nanoparticles	Flame Spray Pyrolysis	5	180	0.1	20	Freundlich, 10.459 mg/g	68

The nanoclay composites, so far have been great adsorbents to remove cationic dyes as reported by^60^.This study has given their potential applicability as absorbents for lead ion removal that could be tried over for removal of other metal ions including, chromium, cadmium, copper, zinc, etc by optimization studies. Further, multisoprtion analysis has showcased the metal removal specificity of the adsorbents for lead.

## Methods

Commercially available bentonite clay, ammonium persulfate, N-acetyl-N,N,N-trimethyl ammonium bromide (CTAB) and sodium dodecyl sulfate were procured from HiMedia. The adsorbents used in the study were Nanoclay, hydrogel and hydrogel composite, which were prepared in the laboratory. Lead, nitric acid, hydrochloric acid and acrylic acid used for hydrogel preparation were obtained from Finar. Millipore distilled water was used for preparation of synthetic lead solution in different concentrations and also for all the other purposes. 5% Nitric acid was used for preparation of lead standard solution to plot standard curve for ICP analysis.

### Preparation of Adsorbents

#### Nanoclay Preparation

Nanoclay adsorbent was prepared using the protocol described by Shrisath *et al*.^61–64^. Briefly, commercially available bentonite was washed 3–4 times and incubated overnight at room temperature. 10 g of weighed clay was added in 100 mL distilled water containing 2 mL HCl. The mixture was heated at 70 °C and then dispersed into 0.5 M CTAB. The resultant was stirred for 12 h at 70 °C and further, dried in oven for 48 h at 80 °C. The dried clay was grinded to get nanoclay powder.

#### Hydrogel preparation

Hydrogel was prepared using by adding 36 mL acrylic acid to distilled water; final volume makeup to 70 mL. Surfactant, Sodium Dodecyl Sulfate (SDS) was added to the solution resulting into acid-SDS-water mixture and kept for sonication at 23 kHz frequency. The initiator was added in a drop wise pattern on reaching 60 °C to the sonicating mixture. The initiator was prepared by addition of 0.3 g Ammonium Per Sulfate (APS) in 5 mL distilled. Gradually, APS initiates the polymerization process by releasing sulfate ions. Further, porosity and swelling rate is maintained by SDS. Polymerized poly(acrylic acid) hydrogel is obtained within 40 minutes of sonication. The procedure has been adopted with a few modifications from Sonawane *et al*.^63^.

#### Nanocomposite preparation

The hydrogel nanocomposite was prepared using nanoclay as the nanoadsorbent/nanofiller. Similar nanocomposite preparation has been conducted by Rafiei *et al*. using different methodology^64^. The protocol for the nanocomposite preparation is unique, where hydrogel preparation was performed by addition of nanoclay in the beginning of sonication process i.e. to the acid-SDS-water mixture. The prepared mixture was sonicated till temperature reaches ~50 °C followed by nanoclay addition in quantities 0.25 g, 0.5 g, 1 g, 1.5 g and 2 g, respectively. The overall percent of nanoadsorbent was varied from 0.25% to 2%.

### Characterization

The prepared bio/nano-adsorbents were characterized by Field Emission Scanning Electron Microscopy (FESEM, Carl Zesis AG) to know the surface morphology. Transmission electron microscopy (TEM, Make: PHILIPS, Model: CM 200) was performed to determine the particle size of nanoclay. Functional group analysis was performed by Perkin-Elmer System 2000 FT-IR spectrophotometer. FT-IR spectrum was obtained by making powdered sample into KBr pellet and was then examined. XRD was used for determination of crystalline material with characteristic peaks in the adsorbents. The adsorbents were scanned, 2θ = 6°–70° in Cu Kα mode with Ni filter. The gallery spacing, d was calculated using Bragg’s Law, given in equation.

1 $$2d\,\sin \,\theta =n\,\lambda $$

where, θ is Bragg’s diffraction angle, *λ* is the Cu K-alpha wavelength (0.15418 nm) and n is a positive integer, here n = 1 due to first order diffraction.

Also, the average crystal size when calculated by Debye-Scherrer equation (Equation) was found to be 12.9333 nm for bulk clay and 9.457 nm for nanoclay.

2 $$D=\frac{K\lambda }{\beta \,\cos \,\theta }$$

where, D is the particle/crystal size, K is the dimensionless constant and a shape factor that is equivalent to 0.9, β is full width at half maximum (FWHM) and θ is Bragg’s diffraction angle.

Thermal analysis of adsorbents were performed using Differential scanning calorimeter (DSC, TA instrument Q200 with T-zero technology) under nitrogen atmosphere with 20 mL/min flow rate. The heating rate was fixed at 10 °C/min and the analysis was continued till 600 °C.

### Batch adsorption studies

The potential nanosorbent preprared in the study was subjected to batch adsorption for lead removal and was followed by optimization of physical and chemical parameters. The shake flask experiments were set up by taking 3 g/L of adsorbent in 250 mL conical flask with 20 mg/L initial lead concentration in the sample, prepared synthetically. The solution was incubated in rotary shaker for 24 hours at room temperature. One-factor at a time method was employed for the optimization study. The effect of pH was studied by varying from 2 to 10 and the remnant lead concentration after incubation was measured using Inductively Coupled Plasma - optical emission spectrometry (ICP-OES). The optimum period of incubation or the contact time was examined by collecting samples at different time intervals from 0 to 24 hours. The readings were taken at fixed intervals of 10–30 minutes for first 2 hours and final reading after 24 hours. While varying one parameter, others were kept constant in each experiment. To optimize the adsorbent dose for 20 mg/L lead concentration, optimized pH and contact time were used. The amount of adsorbent was varied from 0 to 4 g/L. Adorbent dose has to be varied based on the initial lead ion concentration. So, the effect of initial lead ion concentration on optimized adsorbent dose was studied using the optimized parameters (i.e. pH and contact time). The lead concentration at the end of each experiment was measured using ICP (Make: Agilent Technologies). The adsorbent was separated from water sample using centrifugation, where solid particle sediments and supernatant was used for ICP analysis. Stock solution of lead nitrate was prepared by adding 1.5 g (PbNO_3_) in 1L of 5% HNO_3_. Different standards, prepared from the stock, were used for ICP calibration and the unknown concentration of the samples was calculated using prepared calibration curve.

Further, application of nanoclay as filler was studied by experimenting with the nanoclay hydrogel composite. Also, the batch adsorption was conducted in presence of PAA hydrogel as adsorbent to compare the sorption abilities of hydrogel and hydrogel composite. 10 g of each adsorbent variant i.e. hydrogel and composite were added to a column and the study was conducted at optimized pH value. The amount of adsorbent and lead concentration in synthetic water was kept constant. The sample of treated water was collected at regular intervals till the remnant lead concentration reaches equilibrium. The packed bed column after adding adsorbent was placed in the aqueous solution of lead nitrate. The setup was run for certain contact time to achieve a constant remnant lead concentration which was measured using ICP. All the experiments were conducted in triplicates and reported results are an average of each.

### Adsorption kinetics

The study focuses on synthesis and application of Nanoclay, thus, adsorption kinetic study of nanoclay was performed. The rate of the reaction can be determined by pseudo-first order and pseudo-second order equation. The pseudo-first order kinetics was established by Langergren and Svenska in the year 1988 and the rate constant can be calculated by

3 $$\mathrm{log}({q}_{e}-{q}_{t})=\,\mathrm{log}({q}_{e})-(\frac{{k}_{1}}{2.303})(t)$$

where, q_e_ is the amount of lead adsorbed in mg/g at the time of equilibrium, q_t_ the amount of lead adsorbed (mg/g) at time t, k_1_ is the pseudo-first order rate constant (min^−1^) and t is contact time in minutes. The graph is plotted between log (q_e_ − q_t_) and t where the slope helps to determine rate constant. The theoretical q_e_ can be identified by solving the obtained equation for log (q_e_). The experimental q_e_ must be in agreement with theoretical q_e_.

The values of qe and qt can be calculated using following equations:

4 $${q}_{t}=(\frac{{C}_{o}-{C}_{t}}{W})(L)$$

where, C_o_ is the initial amount of lead present in the solution before adsorption in mg/L and C_t_ is the amount of lead in the solution at time t (mg/L) calculated using ICP-OES, W is the amount of adsorbent added (g) and the quantity of the solution in L. Similarly, q_e_ can be calculated by replacing q_t_ by q_e_ and C_t_ by C_e_ in the equation, where, C_e_ is the concentration of lead present in the solution at the time of equilibrium (mg/L).

The pseudo-second order kinetics is to be checked if the pseudo-first order equation doesn’t fit. The equation used for calculation is

5 $$(\frac{t}{{q}_{t}})=(\frac{t}{{k}^{2}\times {{q}_{t}}^{2}})+(\frac{t}{{q}_{e}})$$

where, most of the parameters are same as pseudo-first order equation and k_2_ is the pseudo second-order rate constant (g/mg min).

Further, intra-particle diffusion was also applied to determine the rate kinetics the linear form of the equation used was:

6 $${q}_{t}=({K}_{id}\times {t}^{0.5})+(C)$$

where, K_id_ is the diffusion rate constant and Cis the constant calculated from the graph which was plotted for q_t_ v/s t^0.5^.

### Adsorption isotherms

The isotherm study was performed for the nanosorbent used in the current research. The most commonly used isotherms to determine the type of adsorption are Langmuir and Freundlich isotherms. The linear forms of Langmuir isotherm is defined as:

7 $$\frac{1}{{q}_{t}}=(\frac{1}{{K}_{L}\times {q}_{m}})(\frac{1}{{C}_{e}})+(\frac{1}{{q}_{m}})$$

where, K_L_ is Langmuir constant that defines the affinity of binding sites towards adsorbate (L/mg) and q_m_ stands for maximum adsorption capacity possessed (mg/g). Further, the effective separation can be qualitatively defined by calculating the separation factor, R_L_:

8 $${R}_{L}=(\frac{1}{1+{K}_{L}{C}_{o}})$$

where, C_o_ is the initial lead concentration in mg/L. Langmuir isotherm concentrates more on the homogeneous adsorption of metal ions on adsorbent surface that mostly results in first 10–15 minutes. The rapid adsorption is due to vivid availability of binding sites which reduces over the period of time leading to heterogeneous sorption behavior. The former isotherm is not enough for this type of process and thus, Freundlich isotherm is used in the form of following equation:

9 $$\mathrm{log}({q}_{e})=\,\mathrm{log}({K}_{F})+(\frac{1}{n})\,(\mathrm{log}\,{C}_{e})$$

where, K_F_ is the Freundlich constant that defines the multilayer sorption process owing to chemical and physical adsorption including ion exchange. Also, n^−1^ gives the adsorption intensity and must lie between 0 and 1. Both the parameters can be calculated by the slope of the graph plotted between log q_e_ and log C_e_.

The third isotherm fitted for the present system was Temkin isotherm that determines adsorption capacity and intensity by the constants in the equation:

10 $${q}_{t}=(\frac{RT}{{b}_{T}}){\rm{l}}{\rm{n}}({K}_{T})+(\frac{RT}{{b}_{T}})({\rm{l}}{\rm{n}}({C}_{e})$$

where, b_T_ and K_T_ are related to adsorption intensity (mol/KJ) and adsorption capacity (L/g), respectively. R is the universal gas constant (0.00831 kJ/mol K) and absolute temperature, T, was taken as 298 K. The slope of linear plot between q_e_ and ln C_e_ will help in calculation of b_T_ and K_T_. Lastly, Dubinin-Radushkevich (D-R) model was studied because it is not limited to homogeneous or heterogeneous surface adsorption processes. The linear form of the equation can be stated as:

11 $$\mathrm{ln}({q}_{e})=\,\mathrm{ln}({q}_{m})-\beta {\varepsilon }^{2}$$

where, β is mean free adsorption energy in (mol^2^ K/J^2^), ε is D- R constant. The isotherm is plotted for ln q_e_ v/sε^2^ and ε is calculated using following formula:

12 $$\varepsilon =RT\,\mathrm{ln}(\frac{{C}_{e}+1}{{C}_{e}})$$

The parameters used in the above equation were defined earlier. For mean energy calculation the relation to be applied is:

13 $${E}_{a}=(\frac{1}{\surd (2.\beta )})$$

The constant values are to be calculated from the graph followed by mean energy calculation to differentiate between physical and chemical adsorption. The physical adsorption is defined by the E_a_ value lying between 1–8 KJ/mol.

### Thermodynamics

The thermodynamic parameter Gibbs free energy helps to check the process spontaneity, favorability and stability of the system. So, sorption process carried out in the presence of nanoclay was analyzed in terms of Gibbs free energy (∆G°), calculated as:

14 $${{\rm{\Delta }}}^{\circ }G=-RT\,\mathrm{ln}({K}_{c})$$

15 $${K}_{C}={q}_{e}/{C}_{e}$$

where, K_c_ is thermodynamic equilibrium constant (L/g). Negative value of ∆G° stands for spontaneous reaction whereas, vice versa denotes non-spontaneity in the process.

### Multisorption analysis

The efficient nanoclay hydrogel was subjected to a solution containing six different metal ions to know the selectivity of the adsorbent towards lead. The metal ions used for the study were cobalt, copper, cadmium, chromium, nickel and lead. The divalent ions compete to occupy the number of available active sites. The concentration of the synthetic aqueous solution was kept constant at 20 mg/L and was implied with the optimized set of conditions. The final adsorption analysis was performed by ICP-OES.

### Reusability of adsorbents

The adsorbents, nanoclay and nanoclay hydrogel composite, at the concentration of 4 g/L were saturated with 30 mg/L lead solution for 12 hours. The excess lead ions were washed with distilled water. On drying, the adsorbents were subjected to 0.1 M HCl and 0.1 M NaOH treatment, respectively, for a period of 24 hours. The amounts of lead sequestered in the acidic and basic solution accounted for the percent desorption and was calculated using the formula:

16 $$ \% Desorption=\frac{{C}_{d}.{V}_{1}}{({C}_{o}-{C}_{f})\,{V}_{2}}\times 100$$

where, C_d_ is the final concentration in desorbed solution (mg/L), C_o_ and C_f_ are the initial and final concentrations of the solution prepared for saturating the adsorbent (mg/L), V_1_ and V_2_ are the volume of the desorption and adsorption solution (mL)^34^.

### Statistical Analysis

The optimization data and the adsorption study for single-component and multi-component conducted for nanoclay, hydrogel and hydrogel nanoclay composite, respectively, were carried out in triplicates. Error bars in each graphical representation denotes standard deviation. The significance of data was further analyzed by ANOVA: Single factor, where the level of significance was checked at 1% i.e. P < 0.01 indicating remarkable significance^65^.

### Data Availability

All data generated or analyzed during this study are included in this published article (and its Supplementary Information files).

## Electronic supplementary material

Supplementary information
