# Supplementary material for: Synthesis and implication of novel poly(acrylic acid)/nanosorbent embedded hydrogel composite for lead ion removal
Source: Sci Rep. 2017 Nov 27;7:16413. doi: 10.1038/s41598-017-15642-9 (PMC5703858; doi:10.1038/s41598-017-15642-9)
Supplement: Supplementary file 1 — Supplementary information [file 41598_2017_15642_MOESM1_ESM.doc]

**Synthesis and implication of novel poly(acrylic acid)/nanosorbent embedded hydrogel composite for lead ion removal**

Mayuri Bhatia^a^, Satish Babu Rajulapati*^a^, Shirish Sonawane^b^, Aman Girdher^c^

*^a^ Department of Biotechnology, National Institute of Technology Warangal, Warangal-506004, Telangana, India*

*^b^ Department of Chemical Engineering, National Institute of Technology Warangal, Warangal – 506004, Telangana, India*

*^c^ Department of Biotechnology, Indian Institute of Technology Hyderabad, Sangareddy, Kandi – 502285, Telangana, India*

* Corresponding author. Tel: +91 944 0607238, Fax: +91-870-2459547, E-mail: [satishbabu@nitw.ac.in](mailto:satishbabu@nitw.ac.in)

Effect of pH

The adsorption phenomenon is influenced most by pH. The pH of the solution decides the availability of different cations and anions leading to ionic interaction with metal ions. Lead is a positively charged metal ion, where availability of H^+^ gives a competitive inhibition by occupying the available hydroxyl and carboxyl groups on the active sites. Decrease in H^+^ increases the chances of lead ions to adhere n the active sites of adsorbent. Gradually increase in pH towards alkalinity, enhances the lead hydroxide formation resulting in insolubility and hence, precipitation. The phenomenon has been depicted in figure S1.


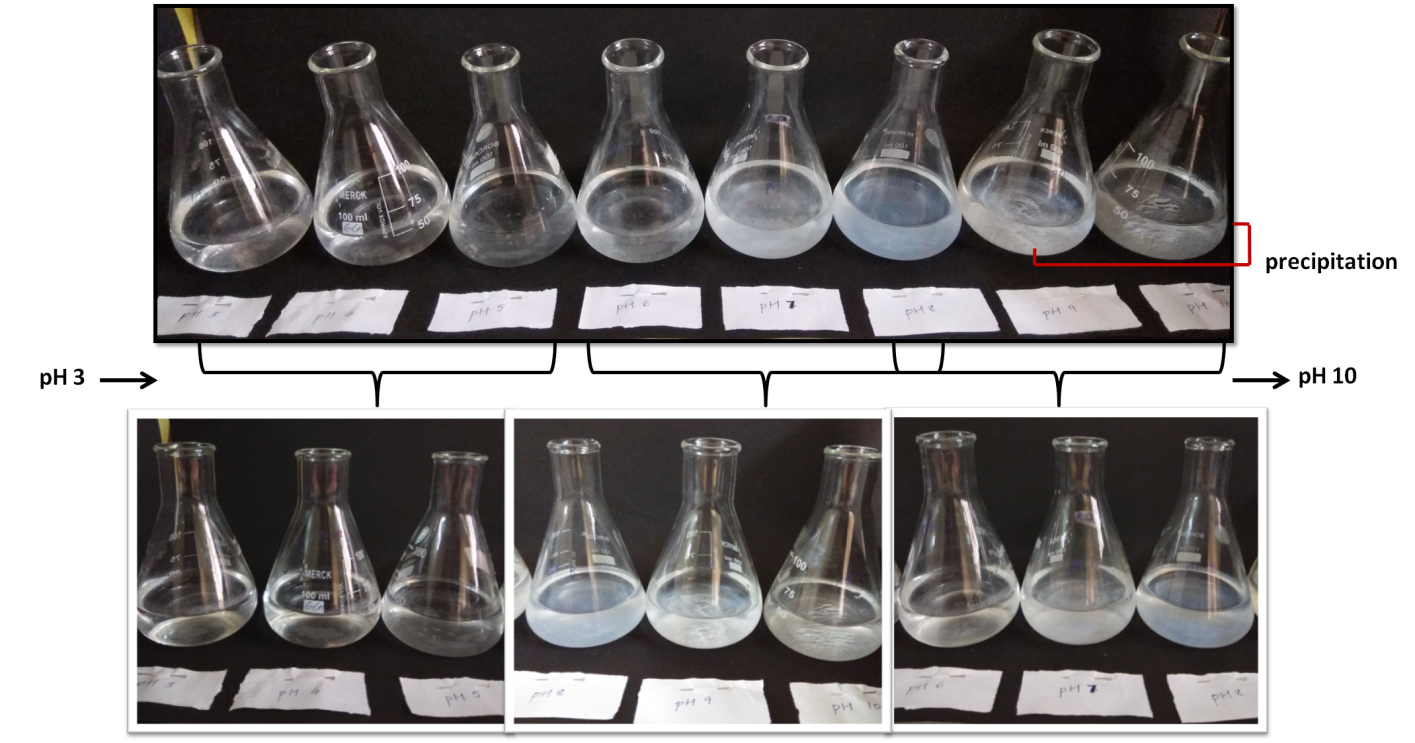


Figure S1: Flask with 20mg/L lead nitrate solution with varying pH from 3.0±0.2 to 10.0±0.2

Adsorption kinetics of Nanoclay

The adsorption mechanism for nanoclay doesn’t follow pseudo-first order kinetics, though the regression coefficient was high. The graph corresponding to the same has been depicted in Figure S2.


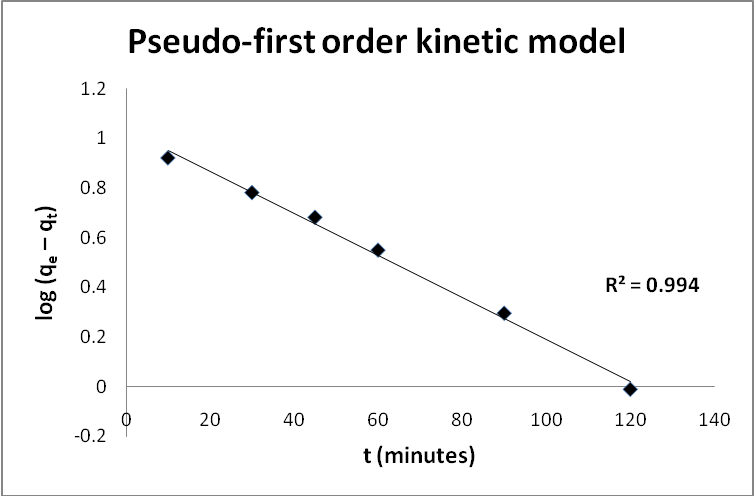


Figure S2: Pseudo-first order kinetic model for nanoclay

Adsorption kinetics of Nanoclay hydrogel composite

The superadsorbent hydrogel used in the study was prepared using acrylic acid as monomer. The polyacrylic acid hydrogel has been reported in literature for dye removal due to high adsorption capacity possessed by the gel ^1^. The graph depicted in figure S2 corresponds to the removal of lead ion attained by 10g of hydrogel in 105 minutes.


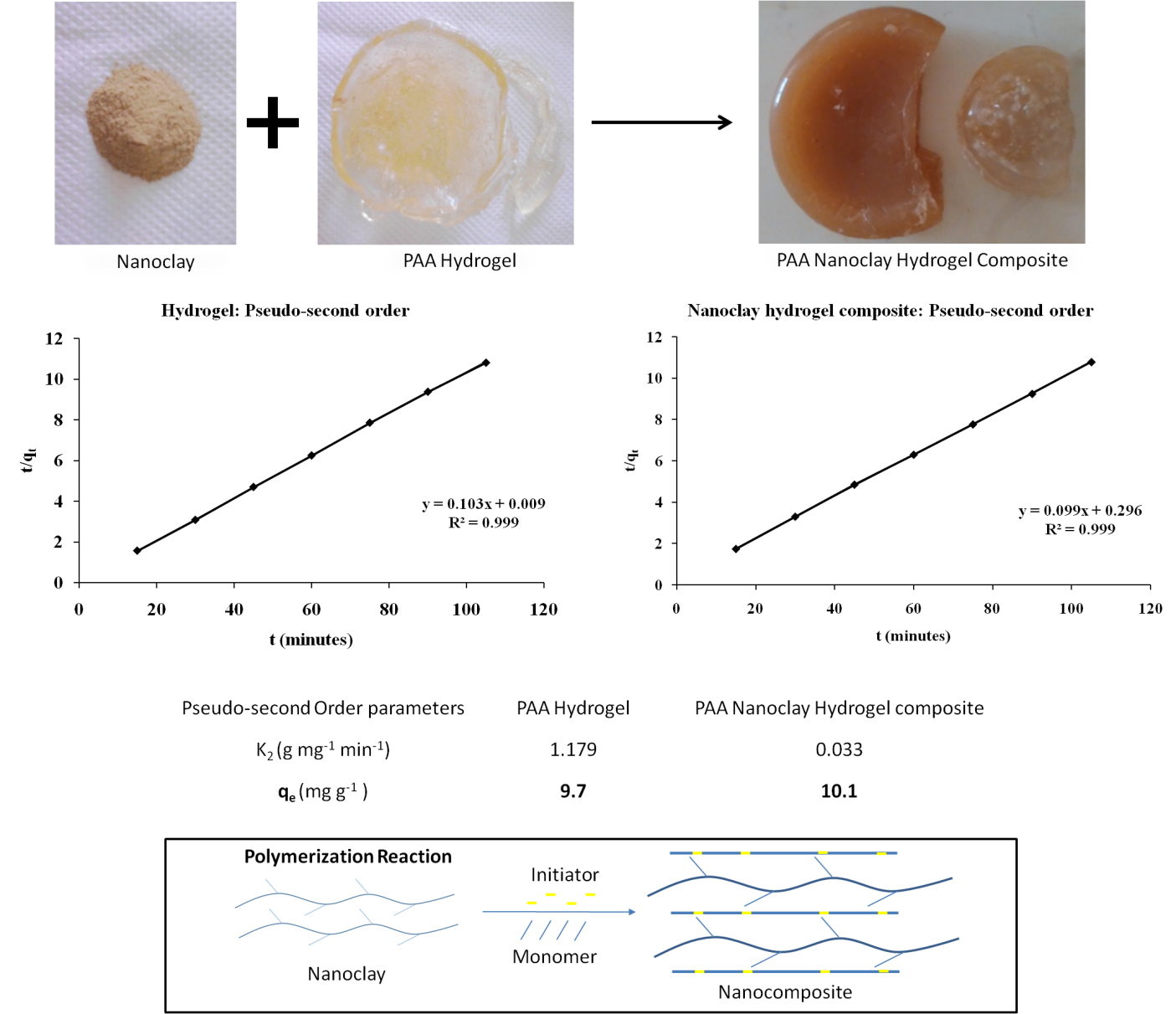


Figure S3: Lead removal by poly acrylicacid (PA) hydrogel, >97% removal achieved in 30 minutes, constant parameters: flow rate = 1.3 ml/min and adsorbent dose = 10g, initial metal ion concentration = 100mg/L; the kinetic study conducted for hydrogel gave 9.7 mg/g adsorption capacity through pseudo-second order kinetic model. On the other hand, nanoclay hydrogel composite was also found to follow similar trend and pseudo-second order kinetic model was observed to be the best fit, where adsorption capacity was found to be better than hydrogel. The polymerization reaction gives an insight of the reaction that occurs to provide binding strength to the polymerizing product i.e. hydrogel nanoclay composite, via nanoclay layers, resulting in increased crosslinking.

1 Kaşgöz, H. & Durmus, A. Dye removal by a novel hydrogel‐clay nanocomposite with enhanced swelling properties*. Polymers for Advanced Technologi*e**s** 19, 838-845 (2008).
